# Prevalence and characterisation of antimicrobial resistance genes and class 1 and 2 integrons in multiresistant *Escherichia coli* isolated from poultry production

**DOI:** 10.1038/s41598-022-09996-y

**Published:** 2022-04-11

**Authors:** Przemysław Racewicz, Michał Majewski, Hanna Biesiada, Sebastian Nowaczewski, Jarosław Wilczyński, Danuta Wystalska, Magdalena Kubiak, Marcin Pszczoła, Zofia E. Madeja

**Affiliations:** 1 Department of Animal Breeding and Product Quality Assessment. Laboratory of Veterinary Public Health Protection, Poznań University of Life Sciences, Poznań, Poland; 2 Department of Genetics and Animal Breeding, Poznań University of Life Sciences, Poznań, Poland; 3 Department of Internal Diseases and Veterinary Diagnostics, Poznań University of Life Sciences, Poznań, Poland; 4 Lab-Vet Laboratory, Tarnowo Podgórne, Poland

**Keywords:** Antimicrobials, Environmental microbiology, Microbial genetics, Public health, Microbial genetics

## Abstract

A global increase in the populations of drug resistant bacteria exerts negative effects on animal production and human health. Our study has been focused on the assessment of resistance determinants in relation to phenotypic resistance of the 74 commensal *E. coli* isolates present in different ecological environments. The samples were collected from poultry litter, feces, and neck skin. Among the microorganisms isolated from the poultry litter (group A), the highest resistance was noted against AMP and DOX (100%). In the *E. coli* extracts from the cloacal swabs (group B), the highest resistance was observed against AMP (100%) and CIP (92%). The meat samples (group C) were characterized by resistance to AMP (100%) and STX (94.7%). Genes encoding resistance to β-lactams (*bla*_TEM_, *bla*_CTX-M_), fluoroquinolones (*qnrA, qnrB, qnrS*), aminoglycosides (*strA-strB, aphA1, aac(3)-II*), sulfonamides (*sul1, sul2, sul3*), trimethoprim (*dfr1, dfr5, dfr7/17*) and tetracyclines (*tetA, tetB*) were detected in the studied bacterial isolates. The presence of class 1 and 2 integrons was confirmed in 75% of the MDR *E. coli* isolates (plasmid DNA), of which 60% contained class 1 integrons, 15% contained class 2 integrons, and 11.7% carried integrons of both classes. Thus, it may be concluded that integrons are the common mediators of antimicrobial resistance among commensal multidrug resistant *Escherichia coli* at important stages of poultry production.

## Introduction

The increasing resistance to commonly applied antimicrobial agents is being reflected by growing multiple drug resistance (MDR) in bacteria and is becoming a growing threat to public health. The use of antimicrobial agents in animal husbandry has been linked to the development and spread of the resistant bacteria^1^. Resistant bacteria can be transferred for example from poultry products to humans via consuming or handling meat contaminated with pathogens^2^. However, the resistance of commensal bacteria is equally important as they constitute a reservoir and vector of resistance determinants in the environment^3^.

Exposure to antimicrobials of different classes can lead to cross-resistance and the selection of antibiotic resistance genes (ARGs) that may spread laterally on mobile genetic elements (MGEs) via horizontal gene transfer (HGT)^4^. HGT is a phenomenon in which genes are transferred between organisms of either the same or different species which often remain in a close ecological relationship^5^.

It has been shown that conjugation is one of the key mechanisms responsible for the spread of the ARGs^6^. One of the most efficient mechanism of acquiring ARGs is facilitated by integrons—a site-specific recombination systems capable of recruiting open reading frames (ORF) in the form of mobile gene cassettes ^7^. Integrons are divided into two distinct subsets, mobile integrons (MIs)—linked to mobile DNA elements, which are primarily involved in the spread of ARGs, and chromosomal integrons (CIs). MIs are associated with conjugation plasmids or transposons (the integron itself is not mobile), which allows the spread and exchange of the resistance genes between individual strains and bacterial species^8^. MIs may be divided into five classes, which are involved in the propagation of antibiotic-resistance genes^9^. These classes are divided based on the sequence of the encoded integrase genes, which share 40–58% sequence homology. The first three classes of integrons are involved in the acquisition of the MDR phenotype. Class 1 integrons are mostly found in clinical and animal production isolates, and most of the known antibiotic-resistance gene cassettes belong to this group. Class 1 is associated with functional and nonfunctional transposons derived from *Tn402* that can be embedded in larger transposons, such as *Tn21*^10^. Class 2 integrons are associated with *Tn*7 derivatives, and class 3 integrons are thought to be located in a transposon inserted in plasmids^11,12^. The integron types may be identified based on the detection of the specific integrase gene sequence—*Int*1, *Int*2 and *Int*3 respectively^13^.

Class 1 and 2 integrons are frequently detected and well characterized, mostly among bacteria belonging to the *Enterobacteriaceae* family, including *E. coli*^14^. The majority of *E. coli* strains are commensals inhabiting the intestinal tract of humans and warm-blooded animals and rarely causes diseases^15^. The adaptation ability of these microorganisms into various niches in host organism is determined by its extremely plastic genome. Another important driving force for the evolution of the *E. coli* genome is the mobile gene pool replaced by the HGT^16^.

Studies of microorganisms found in the breeding environment of broiler chickens (including *E. coli*), such as feces and poultry meat provide valuable information about the reservoir of bacterial genes^17^. While assessing the risks arising from the possible transfer of resistant bacteria within the poultry production chain, it seems important to know the diversity and the prevalence of genetic determinants of antibiotic resistance among the commensal *E. coli* strains.

In the recent years, a number of studies have been carried out aiming to identify the presence and the structure of the integrons, the type of the resistance cassettes and the relationship between the occurrence of integrons and MDR, in commensal and pathogenic *E. coli* isolated from human and animal samples^18–20^. A link between the use of antibiotics in animal production and antimicrobial resistance of human pathogens (within which food is one of the possible vectors) was reported in several studies^21–23^. Nevertheless, little is known about the distribution of integrons in *E. coli* isolated from commercial broiler meat in Poland. Therefore, the purpose of this study was to determine antimicrobial resistance profiles, distribution of class 1 and 2 integrons and integron-associated gene cassettes in commensal strains isolated from poultry litter, broiler chicken feces and meat in western Poland.

## Results

### Antimicrobial resistance phenotypes of E. coli isolates

We have analyzed the incidence of multidrug resistance gene sequences and the prevalence of class 1 and 2 integrons within 74 commensal *E. coli* isolates, obtained from poultry litter (group A, n = 23), swabs from broiler chicken cloaca (group B, n = 26) and poultry meat (group C, n = 25). 60 (81.1%) of them exhibited a multiresistant phenotype (resistance to at least three different antimicrobial agent families). In the first step of study, the phenotypic resistance of the *E. coli* isolates to six antibiotics and chemotherapeutics was assessed.

**Figure 1:**
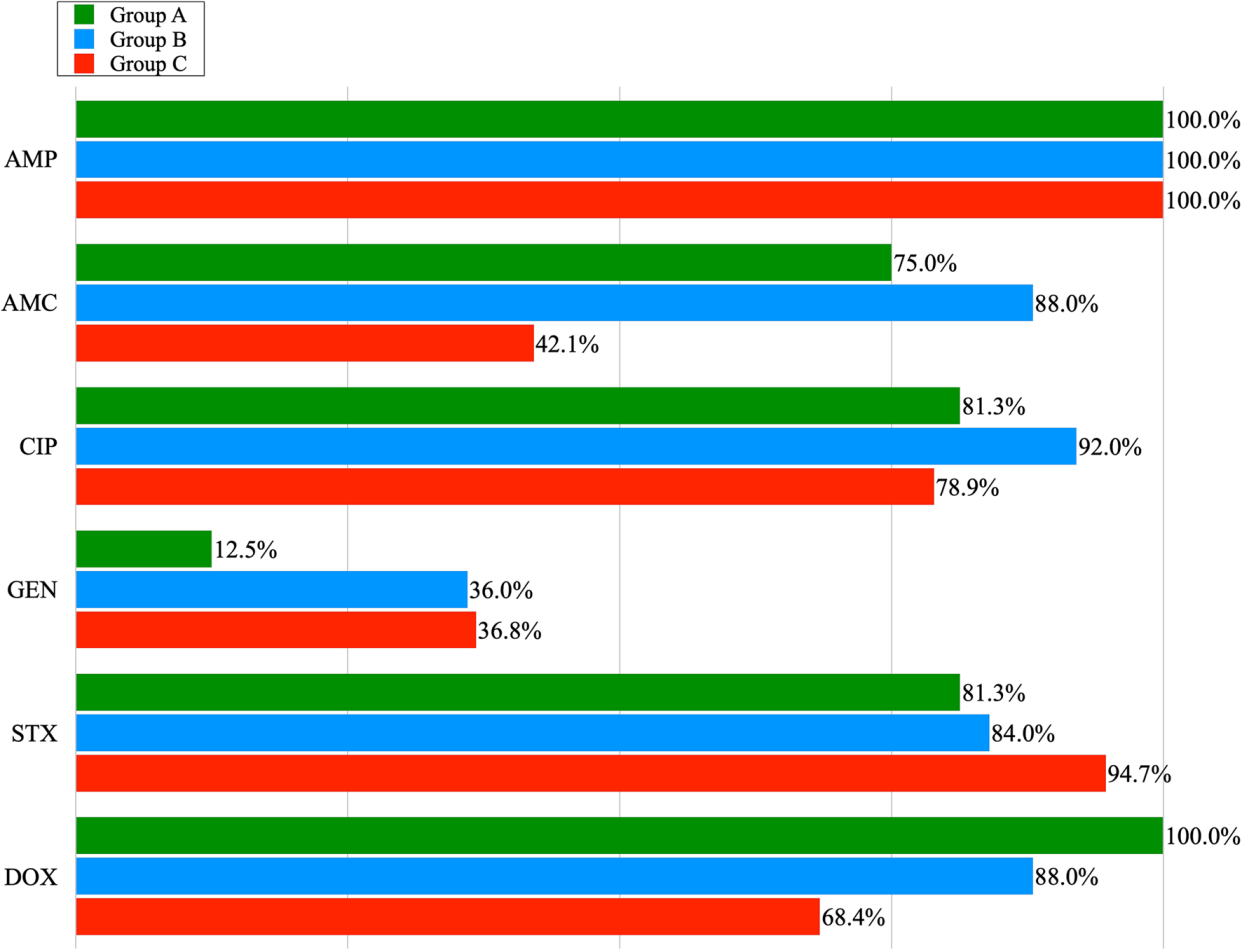
The percentage of phenotypic resistance to 6 antibiotics among the MDR *E. coli* isolates obtained from poultry litter (Group A), cloacal swabs (Group B) and poultry meat (Group C): AMP—Ampicillin; AMC—Amoxycyline with Clavulanic Acid; CIP—Ciprofloxacin; GEN -Gentamicine; STX—Sulfamethoxazole with Trimetoprime; DOX—Doxycycline.

Out of 23 *E. coli* isolates obtained from poultry litter, 16 showed multidrug resistance. The highest resistance was recorded for AMP (100%), DOX (100%), CIP and STX (81.3%), and AMC (75%); the lowest for GEN (12.5%). Out of 26 isolates obtained from chicken cloaca, 25 exhibited MDR. Among the examined MDR isolates, the highest percentage of resistance was observed for the following antibiotics: AMP (100%), CIP (92%), AMC, DOX (88%), STX (84%) and the lowest for GEN (36%). All *E. coli* isolates obtained from the cloaca of chickens, showed phenotypic resistance to the antibiotics classes with which the broilers were treated on farms. Among *E. coli* isolates obtained from meat, 19 of them showed MDR. In this group, the highest resistance was observed for AMP (100%). Lower resistance was noted for: STX (94.7%), CIP (78.9%), DOX (68.4%). Resistance to AMC and GEN was noted in 42.1% and 36.8% of *E. coli* isolates respectively. Figure 1., shows the percentage of phenotypic resistance to 6 antibiotics among MDR *E. coli* isolates obtained from poultry litter, cloacal swabs and poultry meat (group A, B and C). Antimicrobial susceptibility tests showed that 11.7% of all MDR isolates of *E. coli*, were resistant to three antibiotics, 38.3% to four, and 35% showed resistance to five and 15% to six antibiotics (Table 1). Among all *E. coli* isolates positive for integron sequences, the most common drug resistance profile was that of the resistance to: AMP, AMC, CIP, STX, DOX. In the case of 2 isolates with a pair of integrons, *E. coli* isolates that showed resistance to 6 antimicrobial agents were reported. Their resistance profile was the same: AMP, AMC, CIP, GEN, STX, DOX.

**Table 1:** Resistance profiles of multi-drug resistant *E. coli* isolates.

Group	Resistance to 3 antibiotics/chemotherapeutics	Resistance to 4 antibiotics/chemotherapeutics	Resistance to 5 antibiotics/chemotherapeutics	Resistance to 6 antibiotics/chemotherapeutics
	No. of *E. coli* isolates			
A	1	7	7	1
B	1	8	10	6
C	5	8	4	2
Total	7 (11,7%)	23 (38,3%)	21 (35%)	9 (15%)

### Identification and characteristics of resistance genes.

**Figure 2:**
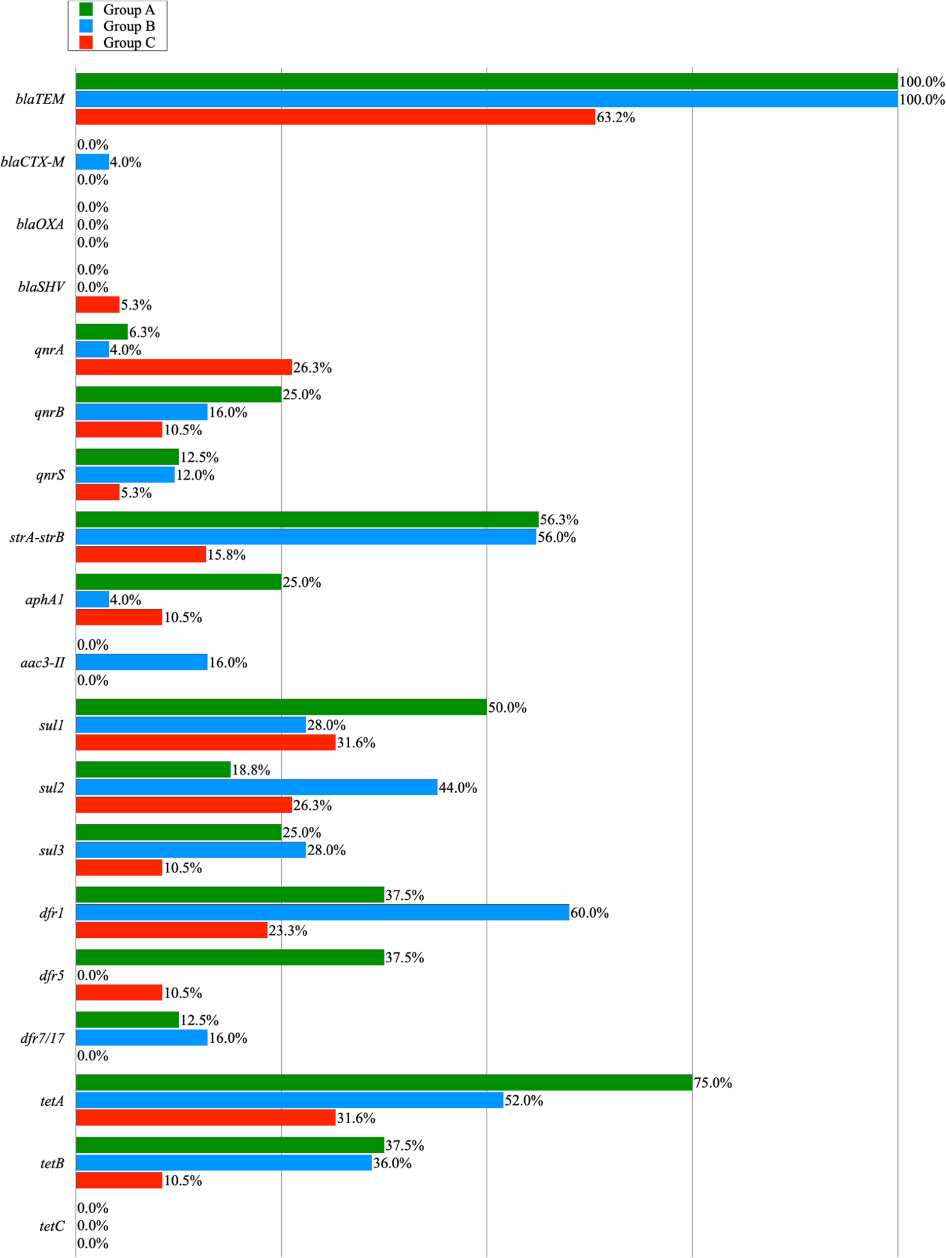
The prevalence of resistance genes among the MRD resistant *E. coli* isolates obtained from poultry litter (Group A), cloacal swabs (Group B) and poultry meat (Group C).

Resistance to AMP and AMC encoded by the narrow spectrum beta lactamase resistance gene (*bla*_TEM_) was found in genomic DNA of all *E. coli* isolates from groups A and B, and in 63,2% of the poultry meat (group C). In case of other genes encoding beta-lactamase resistance, the occurrence of *bla*_CTX-M_ gene was noted in one colon isolate. *Bla*_SHV_ gene was detected in one isolate from the poultry meat swabs. Among the MDR isolates showing the ciprofloxacin (CIP) resistance phenotype, a total of 7 *qnrA* genes, 10 *qnrB* genes and 6 *qnrS* genes were reported in genomic DNA. 18 MDR isolates were gentamicin-resistant, and the *strA-strB*, *aphA1*, and *aac(3)-II* genes, giving resistance to aminoglycosides, were present in: 13 isolates of *E. coli* obtained from litter, 19 isolates from cloacal swabs and 5 isolates from poultry meat. 13 of the 16 tested *E. coli* MDR isolates showed a sulphonamides resistance phenotype which was encoded by: *sul1* (50%), *sul2* (18.8%) and *sul3* (25%). In the case of 21 MDR isolates obtained from cloaca the *sul1* and *sul3* genes were recorded in 28% and the *sul*2 gene in 44% of the cases. In this group, the presence of pairs of *sul* genes was noted in 5 isolates, in the *su1—sul2* combination. In the MDR group of meat isolates, 18 *E. coli* isolates contained the following genes: *sul*1 (31.6%), *sul*2 (26.3%) and *sul3* (10.5%) and in one case a pair of genes (*sul1* and *sul2*) were noted. In the case of *E. coli* isolates recovered from the litter, one of them confirmed 3 genes (*sul1, sul2, sul3*) determining resistance to sulfonamides. Sulfonamide-resistant isolates of *E. coli* showed in most cases compatible resistance to trimethoprim resistance genes (*dfr1, dfr5, dfr7/17*) in 84.6% cases in group A, 71.4% in group B and 38.9% in group C. The *tet* genes (*tetA, tetB*) giving resistance to doxycycline were found in 75 and 37.5% isolates from poultry liter, 52 and 36% isolates from cloacal swab, and 36.8 and 10.5% isolates from meat. The *tetC* gene was not found in any of the studied groups. The pairs of *tetA* and *tetB* genes were found in groups A and B in 4 and 3 cases, respectively. The prevalence of the resistance genes among multidrug resistant *E. coli* isolates obtained from poultry litter (Group A), cloacal swabs (Group B) and poultry meat (Group C) is shown in Table 2 and Fig. 2.

**Table 2:** The prevalence of the resistance genes among multidrug resistant *E. coli* isolates obtained from poultry litter (Group A), cloacal swabs (Group B) and poultry meat (Group C).

	No. of MDR isolates	Number of MDR isolates resistant to a specific antibiotic						Number of MDR isolates where specific resistance genes have been reported																			
		AMP	AMC	CIP	GEN	STX	DOX	*bla*_*TEM*_	*bla*_*CTX-M*_	*bla*_*OXA*_	*bla*_*SHV*_	*qnrA*	*qnrB*	*qnrS*	*strA-strB*	*aphA1*	*aac(3)-II*	*sul1*	*sul2*	*sul3*	*dfr1*	*dfr5*	*dfr7/17*	*tetA*	*tetB*	*tetC*	
Group A	n	16	16	12	13	2	13	16	16	0	0	0	1	4	2	9	4	0	8	3	4	6	6	2	12	6	0
	%		100	75	81.3	12.5	81.3	100	100	0	0	0	6.3	25	12.5	56.3	25	0	50	18.8	25	37.5	37.5	12.5	75	37.5	0
Group B	n	25	25	22	23	9	21	22	25	1	0	0	1	4	3	14	1	4	7	11	7	15	0	4	13	9	0
	%		100	88	92	36	84	88	100	4	0	0	4	16	12	56	4	16	28	44	28	60	0	16	52	36	0
Group C	n	19	19	8	15	7	18	13	12	0	0	1	5	2	1	3	2	0	6	5	2	5	2	0	6	2	0
	%		100	42.1	78.9	36.8	94.7	68.4	63.2	0	0	5.3	26.3	10.5	5.3	15.8	10.5	0	31.6	26.3	10.5	23.3	10.5	0	31.6	10.5	0
Total	n	60	60	42	51	18	52	51	53	1	0	1	7	10	6	26	7	4	21	19	13	26	8	4	31	17	0
	%		100	70	85	30	86.7	85	83.3	1.7	0	1.7	11.7	16.7	10	43.3	11.7	6.7	35	31.7	21.7	43.3	13.3	6,7	51.7	28.3	0

### Detection and characterization of integrons

Our study reports a high incidence of integron-bearing *E. coli* isolates. More than half of the multidrug resistant isolates contained integrons. The presence of class 1 and 2 integrons was confirmed in 45 out of 60 MDR *E. coli* isolates (75%). Structure of 1 class integron, identified by the presence of the *IntI1* gene was detected in 36 (60%) multiresistant isolates in plasmid DNA. Most of this gene was recorded in the DNA of bacteria isolated from cloaca—15 *E. coli* isolates, and next from litter and meat—11 and 10, isolates, respectively. The frequency of the integrons of class 1 was not significantly different across sampling locations (*P* > 0.133). In turn, class 2 integrons, identified by the presence of the intI2 gene, were found in a much smaller number of cases, only in cloacal and meat isolates (4 and 5 cases). The frequency of the integtons of class 2 differed significantly (*P* ≤ 0.05) between these two locations. In the samples in which both classes of the integrons were detected (group B and C), the class 1 integrons were significantly more frequent than class 2 integrons (B: *P* ≤ 0.01, and C: *P* ≤ 0.05).

### Gene cassette analysis of class 1 and 2 integron genes

Table 3, 4 and 5 shows the phenotypes, antibiotic resistance, and prevalence of class 1 and 2 integrons and their resistance gene cassettes in MDR *E. coli* isolates obtained from all groups. *E. coli* isolates containing integrons were grown from poultry litter (group A). 11 out of 16 MDR isolates, contained class 1 integrons only. The variable region of class 1 integrons most frequently contained the *aadA*1 gene cassette in seven cases. The *dfrA1-aadA1* cassette series was present in three cases (18.8%) and *dfrA17-aadA5* in one strain. In the class 2 integron variable region, no gene cassettes were found in any case (Table 3).

**Table 3:** Phenotypes, antibiotic resistance and prevalence of class 1 and 2 integrons and their resistance gene cassettes in MDR *E. coli* isolates obtained from poultry litter. (Group A).

Phenotype of resistance	Mechanisms of resistance	Detection of *intI1*	Genes included in cassettes	Detection of *intI2*	Genes included in cassettes
	Resistance genes detected				
AMP, AMC, CIP, GEN, STX, DOX	*bla*_*TEM*_, *qnrB, sul1, dfr1, tetA*	+	*aadA1*		
AMP, AMC, CIP, STX, DOX	*bla*_*TEM*_, *qnrS, strA-strB, aphA1, sul1, dfr5, dfr7/17, tetA,tetB*	+	*dfrA17-aadA5*		
AMP, AMC, CIP, STX, DOX	*bla*_*TEM*_, *qnrS, strA-strB, aphA1, sul1, dfr1, dfr5, tetA, tetB*	+	*dfrA1- aadA1*		
AMP, AMC, CIP, STX, DOX	*bla*_*TEM*_, *strA-strB, sul1, sul2, dfr1, tetA*	+	*dfrA1- aadA1*		
AMP, AMC, CIP, STX, DOX	*bla*_*TEM*_, *strA-strB, sul2, dfr5, tetB*	+	*aadA1*		
AMP, AMC, CIP, STX, DOX	*bla*_*TEM*_, *qnrB, strA-strB, sul1, sul2, sul3, dfr1, tetA, tetB*	+	*aadA1*		
AMP, AMC, CIP, STX, DOX	*bla*_*TEM*_, *strA-strB, aphA1, dfr5, tetA*				
AMP, AMC, CIP, STX, DOX	*bla*_*TEM*_, *qnrA, qnrB*				
AMP, AMC, STX, DOX	*bla*_*TEM*_, *sul1, sul3, dfr1, tetA*	+	*aadA1*		
AMP, CIP, STX, DOX	*bla*_*TEM*_, *strA-strB, sul1, dfr1, tetA*	+	*dfrA1- aadA1*		
AMP, CIP, STX, DOX	*bla*_*TEM*_, *dfr5, tetA, tetB*	+	*aadA1*		
AMP, CIP, STX, DOX	*bla*_*TEM*_, *strA-strB, aphA1, sul1, dfr7/17, tetB*	+	*aadA1*		
AMP, AMC, CIP, DOX	*bla*_*TEM*_, *tetA*				
AMP, AMC, CIP, DOX	*bla*_*TEM*_, *tetA, aadA1*				
AMP, AMC, GEN, DOX	*bla*_*TEM*_, *sul3*				
AMP, STX, DOX	*bla*_*TEM*_, *qnrB, strA-strB, sul3, dfr5, tetA*	+	*aadA1*

**Table 4:** Phenotypes, antibiotic resistance and prevalence of class 1 and 2 integrons, detected in 25 MDR *E. coli* isolates obtained from cloacal swabs (Group B).

Phenotype of resistance	Mechanisms of resistance	Detection of *intI1*	Genes included in cassettes	Detection of *intI2*	Genes included in cassettes
	Resistance genes detected				
AMP, AMC, CIP, GEN, STX, DOX	*bla*_*TEM*_, *qnrB, qnrS, sul1, dfr1, tetB*	+	*dfrA5*	+	*dfrA1-sat2- aadA1*
AMP, AMC, CIP, GEN, STX, DOX	*bla*_*TEM*_, *qnrB, qnrS, dfr1, tetA*	+	*aadA1*	+	*dfrA1-sat2- aadA1*
AMP, AMC, CIP, GEN, STX, DOX	*bla*_*TEM*_, *strA-strB, aac3-2a, sul1, dfr1, tetA*	+	*dfrA1- aadA1*		
AMP, AMC, CIP, GEN, STX, DOX	*bla*_*TEM*_, *strA-strB, aac3-2a, sul1, sul2, dfr7/17, tetB*	+	*dfrA17-aadA5*		
AMP, AMC, CIP, GEN, STX, DOX	*bla*_*TEM*_, *sul3, dfr1*	+		+	*dfrA1-sat2- aadA1*
AMP, AMC, CIP, GEN, STX, DOX	*bla*_*TEM*_, *qnrB, sul2, dfr1*	+			
AMP, AMC, CIP, STX, DOX	*bla*_*TEM*_, *sul1, sul2, dfr1, tetA*	+	*dfrA5*		
AMP, AMC, CIP, STX, DOX	*bla*_*TEM*_, *strA-strB, sul3, dfr1, dfr7/17, tetB, aadA1*	+	*aadA1*		
AMP, AMC, CIP, STX, DOX	*bla*_*TEM*_, *qnrA, sul1, sul2, dfr1*	+	*aadA1*		
AMP, AMC, CIP, STX, DOX	*bla*_*TEM*_, *dfr1, tetA*	+		+	*dfrA1-sat2- aadA1*
AMP, AMC, CIP, STX, DOX	*bla*_*TEM*_, *sul3, tetA*				
AMP, AMC, CIP, STX, DOX	*bla*_*TEM*_, *qnrB, qnrS, strA-strB, sul2, dfr1, tetA*				
AMP, AMC, CIP, STX, DOX	*bla*_*TEM*_, *strA-strB, sul3, dfr1, tetB*				
AMP, AMC, CIP, STX, DOX	*bla*_*TEM*_, *sul3, dfr1, tetA*				
AMP, AMC, CIP, GEN, DOX	*bla*_*TEM*_, *qnrS, strA-strB, aac3-2a tetA*	+	*aadA1*		
AMP, AMC, CIP, GEN, DOX	*bla*_*TEM*_, *strA-strB, tetA, tetB*				
AMP, AMC, CIP, STX	*bla*_*TEM*_, *blaCTX-M, sul1, sul2, dfr1*	+	*dfrA1- aadA1*		
AMP, AMC, CIP, STX	*bla*_*TEM*_, *strA-strB, sul2*				
AMP, AMC, CIP, STX	*bla*_*TEM*_, *qnrS, strA-strB, sul2*				
AMP, AMC, CIP, DOX	*bla*_*TEM*_, *qnrS, strA-strB, aac3-2a, tetA*				
AMP, AMC, STX, DOX	*bla*_*TEM*_, *dfr1, tetA*	+	*dfrA1- aadA1*		
AMP, CIP, STX, DOX	*bla*_*TEM*_, *strA-strB, aphA1, sul1, sul2, tetA, tetB*	+	*dfrA1- aadA1*		
AMP, CIP, STX, DOX	*bla*_*TEM*_, *strA-strB, sul2, dfr7/17, tetB*				
AMP, CIP, STX, DOX	*bla*_*TEM*_, *strA-strB, sul2, dfr7/17, tetB*	+			
AMP, AMC, DOX	*bla*_*TEM*_, *qnrB, strA-strB, tetA, tetB*

**Table 5:** Phenotypes, antibiotic resistance and prevalence of class 1 and 2 integrons, detected in 19 MDR *E. coli* isolates obtained from meat (Group C).

Phenotype of resistance	Mechanisms of resistance	Detection of *intI1*	Genes included in cassettes	Detection of *intI2*	Genes included in cassettes
	Resistance genes detected				
AMP, AMC, CIP, GEN, STX, DOX	*bla*_*TEM,*_*qnrB, qnrS, strA-strB, aphA1, sul1, sul3, dfr1, tetB*	+	*dfrA1- aadA1*		
AMP, AMC, CIP, GEN, STX, DOX	*bla*_*TEM*_, *strA-strB, sul1, tetA*	+	*dfrA1- aadA1*	+	
AMP, AMC, CIP, STX, DOX	*bla*_*TEM*_, *strA-strB, sul1, dfr5*	+	*aadA1*		
AMP, AMC, CIP, STX, DOX	*bla*_*TEM*_, *aphA1, sul3, dfr1*	+		+	
AMP, CIP, GEN, STX, DOX	*bla*_*TEM,*_*sul1, tetB*	+	*dfrA1- aadA1*		
AMP, CIP, GEN, STX, DOX	*bla*_*TEM*_, *qnrA, strA-strB, sul1, dfr1, tetA*	+	*dfrA1- aadA1*		
AMP, AMC, STX, DOX	*bla*_*TEM*_, *qnrB, qnrS, strA-strB, sul1, sul2, dfr1, tetA*	+	*dfrA1- aadA1*	+	
AMP, AMC, STX, DOX	*bla*_*TEM*_, *sul2, tetA*			+	*dfrA1-sat2-aadA1*
AMP, CIP, STX, DOX	*bla*_*TEM*_, *sul1, tetA*	+	*dfrA1- aadA1*		
AMP, CIP, STX, DOX	*bla*_*TEM*_, *strA-strB, sul2*	+	*aadA1*		
AMP, CIP, STX, DOX	*bla*_*TEM*_, *strA-strB, sul2, dfr5, tetA*				
AMP, CIP, STX, DOX	*qnrA*				
AMP, AMC, CIP, STX	*blaSHV, qnrA*				
AMP, AMC, CIP, GEN	*qnrB, aphA1*				
AMP, CIP, STX	*qnrA*			+	*dfrA1-sat2-aadA1*
AMP, CIP, STX	*bla*_*TEM*_, *tetA*				
AMP, CIP, STX	*qnrA*				
AMP, STX, DOX		+	*dfrA1- aadA1*		
AMP, GEN, STX	*qnrA*				

In case of the multidrug-resistant *E. coli* isolates obtained from cloacal swabs from broiler chickens (group B), 15 isolates contained class 1 integrons and 4 isolates contained class 2 integrons (Table 4). The results of DNA sequencing of the inserted gene cassettes allowed to identify 11 class 1 integrons containing 5 different gene cassettes: *aadA1* (36.4%), *dfrA5* (18.2%) and arrays of cassettes: *dfrA1-aadA1* (36.4%) and *dfrA17-aadA5* (9.1%) in five separate isolates. Genes contained in the four class 2 integron cassettes contained gene cassette arrays: *dfrA1-sat2-aadA1*.Two isolates containing class 1 and 2 integrons contained cassette arrays: *aadA1; dfrA1-sat2-aadA1* and *dfrA5; dfrA1-sat2-aadA1*. Four (26.7%) class 1 positive integron isolates had no gene cassettes in their variable part.

In the group of MDR *E. coli* isolated from the poultry meat (group C), genes contained in the class 1 integron variable region were detected in 9 cases (Table 5). VR class 1 integrons showed less variation compared to group B isolates and usually contained 2 gene cassette arrays: *aadA1* (22.2%) and *dfrA1-aadA1* (77.8%). The variable region of 5 class 2 integrons contained in only two cases a set of cassettes: *dfrA1-sat2-aadA1*. In one strain, a pair of class 1 and 2 integrons was detected as well as their variable parts were empty.

## Discussion

It is estimated that in most developed nations, livestock use 50–80% of antibiotics produced^24^. Commonly used groups of veterinary medicinal products in the EU are tetracyclines (32%), penicillins (26%), sulfonamides (12%), macrolides (7%), polymyxins (5%) and aminoglycosides (5%)^25,26^.

In Poland, 829 tones of antibiotics are used annually, of which as much as 578 tones in the agricultural industry^27,28^. The most commonly used groups of substances in years 2014–2016 were tetracyclines (42.34–49.07%), penicillins (18.98–23.40%) and macrolides (11.69–13.22%)^26^.

In our study, 74 unrelated commensal isolates of *Escherichia coli* originated from poultry litter, cloacal swabs and poultry meat were phenotypically and genotypically tested for the antimicrobial resistance and the presence of integrons as factor, for the development of antibiotic resistance and the emergence of MDR strains. Among them, 60 isolates (81.1%) were multiresistant (resistant to a minimum of three classes of antibiotics). We have found the highest level of antimicrobial resistance (96.2%) in the *E. coli* isolates obtained from broiler intestinal swabs (group B).

The high incidence of multidrug resistance in our study, particularly regarding isolates obtained from feces and meat, is extremely significant and should be regarded as a serious health risk due to the fact, that multidrug resistant isolates may have a chance of contaminating food products, and consequently being transferred to humans.

The percentage of resistance to some antimicrobial agents (Ampicillin, Doxycycline, Trimetophrim, Sulfamethoxazole, and Ciprofloxacin) in all studied groups was particularly high (100–68.4%), which indicates that the commensal *E. coli* isolates may be a reservoir of resistance to antibiotics and chemotherapeutics. Our data largely overlaps with the data made available by the European Food Safety Authority and the European Centre for Disease Prevention and Control, on the resistance profile of the commensal *E. coli* isolates obtained from slaughterhouse broilers, collected between 2009 and 2014 in Poland^28^. It confirms high resistance of *E. coli* isolates to nalidixic acid ciprofloxacin, and ampicillin (70–90%) and a limited resistance to tetracyclines, sulfonamides and streptomycin^28,29^.

The *bla*_TEM_ gene encoding β lactamase, which gives resistance to penicillins and cephalosporins of the first generation, has been detected in all multidrug-resistant isolates obtained from litter and feces. The dominance of the *TEM* gene over the *CTX* gene in fecal poultry isolates was also noted in the Nigerian^30^ study but was not as pronounced as in our experiment (63%-*TEM* and, 35% *CTX-M*). Although *E. coli* isolates with enzymes belonging to the CTX-M family in ESBL-positive bacteria are currently predominant in the world^31^ in our study the *bla*_CTX-M_ gene was reported only in one bacterial strain (4%) from cloacal swabs. Similar results were obtained in China, where *bla*_CTX-M_ was detected in 1.6% of the isolates coming from the meat^32^.

Quinolone resistance is a current worldwide problem in human and veterinary medicine^33^. Quinolone resistance can encoded in bacterial chromosome or be present in plasmids. Plasmid-mediated quinolone resistance (PMQR) promote the spread of the multi-drug resistance phenotype. For example, *qnr* genes present on MDR plasmids are often found with genes encoding β-lactamases^34^. Of all Qnr determinants present in the our study, the *qnrB* gene was found most frequently. Similar results were published in other studies^33,35^. The occurrence of PMQR is also associated with resistance to other groups of antibiotics. The mechanism responsible for this phenomenon is related to the presence of the aminoglycoside acetyltransferase enzyme—AAC(6')-Ib-cr, which modifies both the molecular structure of some fluoroquinolones and aminoglycosides or the *oqxAB* gene encoding an MDR-type efflux pump contributing to increased resistance to quinolones and chloramphenicol, trimethoprim and quinolones^36,37^. In Seo and Lee^33^ study, 10 PMQR-positive *E. coli* were isolated from chicken meat, and these isolates also showed higher resistance rates to several antimicrobial agents when compared to PMQR-negative *E. coli*. This is consistent with previous studies showing that the PMQR genes increase resistance to other antimicrobials and cause MDR to drugs such as: aminoglycosides, β-lactams, chloramphenicol, sulfonamides, tetracyclines and trimethoprim^38^.

Tetracyclines are commonly used to treat bacterial infections in livestock, including poultry in many countries^39–41^. Due to the numerous advantages of tetracyclines, such as their widespread availability, low cost, and several side effects, the use of such antibiotics to treat animal and human infections has been increasing in recent years^42^. The chickens are treated with tetracycline orally and their metabolites (up to 90%) are excreted in the feces^43^ on manure^44^. It is noteworthy that in our study the highest resistance to doxycycline was recorded among *E. coli* isolates derived from poultry litter, which is a mixture of poultry manure, litter, feathers, feed, and spilled drinking water that accumulates during breeding^45^. However, the proportion of *E. coli* isolates with resistance to tetracyclines was lower than the proportion of *E. coli* isolates resistant to beta lactam antibiotics. Similar results were obtained in study by Islam et al.^46^ in which MDR isolates were the most resistant to tetracyclines (96.6%) and penicillins (100%). In addition to the antibiotic residues, manure also contains MDR bacteria and resistance genes, which can be transmitted to humans through direct contact between poultry and humans or indirectly via the food chain^47^. The results of genotyping showed that similarly to other published data, the resistance of commensal *E. coli* to tetracyclines was induced by the presence of *tetA* and *tetB* genes^48^. The highest content of *tet* genes in poultry litter isolates confirms the thesis put forward by Furtula et al.^44^, that the breeding environment significantly contributes to the spread of the resistance, via the transmission of the resistance genes.

Resistance to sulfonamides in Gram-negative bacteria generally results from the presence of the genes *sul1*, *sul2*, and/or *sul3*. Among them, the *sul2* gene is the most widely distributed *sul* gene in porcine, avian, or human *E. coli* isolates, and it plays an important role in sulfonamide resistance^49^. In our research, the prevalence of *sul2* genes was highest in isolates from cloaca swabs (44%) and was similar to the results of other studies^50,51^. Interestingly, in only one strain obtained from litter, all tested *sul* genes were determined. The frequency of *sul* genes detection in our experiment corresponded to other studies^50,51^. The selective pressure exerted by sulfonamides in the poultry industry appears to be high, which may favor the maintenance of acquired *sul* genes among bacteria^52^.

In our study, the total prevalence of integrons (75%) in MDR isolates was higher than the prevalence of integrons detected in other poultry production prevalence studies^53,54^. We also found a clear predominance of class 1 integrons in relation to class 2 integrons, which is consistent with previous studies that also showed the highest prevalence of this class in poultry isolates^55,56^.

Most of the integrons detected in our study contained gene cassettes encoding resistance to trimethoprim (*dfrA* gene type) and streptomycin/spectinomycin belonging to aminoglycosides (*aadA* gene type), and the most frequently detected sequence of cassettes was *dfrA1-aadA1*. The persistence of these genes, which have been reported worldwide in isolates from different sources, may be related to the widespread use of streptomycin/spectinomycin, trimethoprim, sulfonamides and other antibiotics in food producing animals. Although the afore-mentioned aminoglycosides are not used therapeutically in animals in Poland, the presence of *aadA* genes may be a form of genetic memory, in case of re-exposure of the microorganism to this group of antibiotics^57^.

The analysis of the variable part of integrons in our experiments indicated the presence of one to three gene cassettes. In group B, we noted the highest number of integrons among all tested groups and a greater variety of gene cassettes within the integron variable part. The higher prevalence of class 1 integrons among the *E. coli* isolates obtained from the cloaca may be caused by the development and spread of the resistance genes, due to the misuse or abuse of antibiotics in the poultry production^1^.

Of all MDR *E. coli* isolates that had integrons, 8 isolates (13.3%) did not contain any of the evaluated gene cassettes. The situation regarding the so-called "empty integrons" has already been described by Fonseca et al.^58^, where it was indicated that these bacteria could rapidly develop into MDR in the future. However, it cannot be ruled out that integrons may have previously removed cassettes of resistance genes acquired by cutting them out for unknown reasons^59^.

The data obtained in this study highlights the importance of commensal *E. coli* in the spread of resistance genes at different stages of poultry production. We confirmed that more than half of the multidrug-resistant isolates (75%) contained integrons. Furthermore, we showed that antibiotic resistance can also occur on non-integron structures. Therefore, there is a need for further detailed genetic studies on the evolution of isolates present in poultry to uncover the underlying mechanisms the acquisition of resistance by these microorganisms and to analyze the implications for humans. Such data may be used to determine the dynamics of resistance development and strategies to counteract antibiotic resistance among zoonotic microorganisms transmitted through food of animal origin at all stages of the food chain, from farm to table.

## Materials and methods

### E. coli isolates

A total number of 74 *E. coli* isolates was collected from three areas of poultry production: litter swabs from chicken houses (n = 23), cloacal swabs from chicken (n = 26), chicken meat from slaughterhouses (n = 25). All samples were collected between November 2019 and March 2020.

Litter samples from 23 different chicken houses were acquired in accordance with the boot swabs sampling procedure guidelines of the national control program for *Salmonella* serotypes in poultry flocks in line with the guidelines of the current EU law^60^. Samples were collected from 4-week-old chicks (average weight 1.6 kg) 2 weeks before slaughter. The samples of cloacal swabs were collected using swabs (NRS II Transwab swabs with 10 Buffered Peptone Water, Medical Wire & Equipment, Corsham, United Kingdom) in a poultry slaughterhouse. Chickens were raised on 25 unrelated farms located in Greater Poland Voivodship. Birds that were sent to slaughter at 6 weeks of age, weighed an average of 3 kg and belonged to the Ross 308 breed. The chicken meat samples were obtained from neck skin. These samples were delivered to the laboratory for testing, as part *Salmonella* monitoring program, from 5 different poultry slaughterhouses located in the Greater Poland Voivodeship, from different periods of production.

Information regarding the antibiotics used in the above chickens (name of the antibiotic, withdrawal periods) was included in the food chain documentation. In the treatment of poultry, the most frequently used antibiotics were: Amoxicillin, Enrofloxacin, Doxycycline, Sulfamethoxazole / Trimethoprim.

The samples were placed in buffered peptone water (BioMerieux, Marcy l'Etoile, France) and incubated at 35 °C (± 1 °C) for 18 h (± 2 h) under aerobic conditions. Next, the material was plated on MacConkey agar medium (OXOID, Basingstoke, United Kingdom) and incubated for 24 ± 2 h under aerobic conditions at 37 °C ± 1 °C. Colonies with the typical *E. coli* phenotype were selected (one per sample) and verified by the MALDI-TOF method (Bruker, Bremen, Germany). The score ranged from 2.015 to 2.152.

### Antimicrobial susceptibility testing

Antibiotic susceptibility tests of all 74 *E. coli* isolates were performed following the standard agar disk diffusion method, according to the CLSI (Clinical and Laboratory Standards Institute-2012) using commercially available antimicrobial disks containing (OXOID, Basingstoke, United Kingdom): penicillins (Ampicillin—AMP 10 μg and Amoxycyline with Clavulanic acid—AMC 20/10 μg), fluoroquinolones (Ciprofloxacin—CIP 5 μg), aminoglycosides (Gentamicine—GEN 10 μg), sulfonamides (Sulfamethoxazole with Trimetoprime—STX 25 μg), tetracyclines (Doxycycline—DOX 30 μg).

The following media were used for the tests: Mueller Hinton Broth (Thermo Fisher Scientific, Waltham, Massachusetts, USA), Mueller–Hinton agar (OXOID, Basingstoke, United Kingdom). The bacterial colonies were classified as sensitive, intermediate, or resistant according to the standardized CLSI guidelines (VET 01S—Performance standards for antimicrobial disk and dilution susceptibility tests for bacteria isolated from animals) and *E. coli* ATCC 25922 strain was used as control. The isolates were collected and stored at -80℃ for further analyses on Viabank medium (OXOID, Basingstoke, United Kingdom).

### Detection of integrons

Class 1 and Class 2 integrons were detected based on the presence of gene sequences characteristic for integrase 1 *(IntI1*) and integrase 2 (*IntI2*) respectively. Selected regions were amplified by qualitative PCR carried out on plasmid DNA extracted from the three studied groups (poultry litter, feces and carcasses).

Plasmid DNA from the bacterial samples was extracted with the Gene Matrix Plasmid Miniprep DNA Purification Kit (E3500 EurX, Gdansk, Poland) according to the manufacturer’s protocol. PCR amplification was done in a 10µL mixture containing: 1µL DNA template, 0.3µL of primers (0.3 μM), 2µL of 5× HOT FIREPol® Blend Master Mix kit (04-25-00S25, SolisBiodyne, Tartu, Estonia) and molecular biology graded water (nuclease free, W4502 Merck, Darmstadt, Germany). The thermal cycling conditions included: preincubation at 95 °C (15 min), followed by 38 cycles of denaturation at 95 °C (20 s), annealing at 61 °C (45 s), extension at 72 °C (60 s) and final extension at 72 °C (5 min). Primer pair sequences are listed in Table 6. The specificity of the PCR reaction (product length–base pair, bp) was verified by electrophoresis on a 1.5% agarose gel.

**Table 6:** Primer pairs used for PCR analyses in this study.

Group of antibiotics	Target gene		Primer sequence	Product length (bp)	Reference
beta-lactams	*bla*_*SHV*_	F	CTTTATCGGCCCTCACTCAA	237	Fang et al.^61^
		R	AGGTGCTCATCATGGGAAAG		
	*bla*_*TEM*_	F	CGCCGCATACACTATTCTCAGAATGA	445	
		R	ACGCTCACCGGCTCCAGATTTAT		
	*bla*_*CTX-M*_	F	ATGTGCAGYACCAGTAARGTKATGGC	593	
		R	TGGGTRAARTARGTSACCAGAAYCAGCGG		
	*bla*_*OXA*_	F	ACACAATACATATCAACTTCGC	813	
		R	AGTGTGTTTAGAATGGTGATC		
Fluoroquinolones	*qnrA*	F	ATTTCTCACGCCAGGATTTG	529	Marti et al.^62^
		R	GCAGATCGGCATAGCTGAAG		
	*qnrB*	F	GGMATHGAAATTCGCCACTG	429	
		R	TTYGCBGYYCGCCAGTCGAA		
	*qnrS*	F	GACGTGCTAACTTGCGTGAT	393	
		R	TGGCATTGTTGGAAACTTG		
Aminoglycosides	*strA-strB*	F	ATGGTGGACCCTAAAACTCT	893	Kozak et.al.^63^
		R	CGTCTAGGATCGAGACAAAG		
	*aphA1*	F	ATGGGCTCGCGATAATGTC	600	Kozak et.al.^63^
		R	CTCACCGAGGCAGTTCCAT		
	*Aac(3)-II*	F	ATATCGCGATGCATACGCGG	877	Hu et al.^64^
		R	GACGGCCTCTAACCGGAAGG		
Sulfonamides	*sul1*	F	CGGCGTGGGCTACCTGAACG	433	Kozak et.al.^63^
		R	GCCGATCGCGTGAAGTTCCG		
	*sul2*	F	CGGCATCGTCAACATAACCT	721	
		R	TGTGCGGATGAAGTCAGCTC		
	*sul3*	F	CAACGGAAGTGGGCGTTGTGGA	244	
		R	GCTGCACCAATTCGCTGAACG		
Trimethoprim	*dfr1*	F	TGGTAGCTATATCGAAGAATGGAGT	425	Grape et al.^65^
		R	TATGTTAGAGGCGAAGTCTTGGGTA		
	*dfr5*	F	AGCTACTCTTTAAAGCCTTGACGTA	341	
		R	GTGTTGCTCAAAAACAACTTCG		
	*dfr7/17*	F	ACATTTGACTCTATGGGTGTTCTTC	280	
		R	AAAACTGTTCAAAAACCAAATTGAA		
Tetracyclines	*tetA*	F	GGCGGTCTTCTTCATCATGC	502	Kozak et.al.^63^
		R	CGGCAGGCAGAGCAAGTAGA		
	*tetB*	F	CGCCCAGTGCTGTTGTTGTC	173	
		R	CGCGTTGAGAAGCTGAGGTG		
	*tetC*	F	GCTGTAGGCATAGGCTTGGT	888	
		R	GCCGGAAGCGAGAAGAATCA		
Class 1 integrase	*IntI1*	F	GCCTTGCTGTTCTTCTACGG	565	Levesque, et al.^67^
		R	GATGCCTGCTTGTTCTACGG		
Class 2 integrase	*IntI2*	F	AAGCAGACTTGACCTGA	565	White, et al. 2001^68^
		R	CACGGATATGCGACAAAAAGGT		
Class 1 integron cassette	*Integron CL_F*	F	GGCATCCAAGCAGCAAG	Variable	Levesque et al.^67^
	*Integron CL_R*	R	AAGCAGACTTGACCTGA		
Class 2 integron cassette	*Integron CL_II_F*	F	CGGGATCCCGGACGGCATGCACGATTTGTA	Variable	White et al.^68^
	*Integron CL_II_R*	R	GATGCCATCGCAAGTACGAG		

### Sequencing of the variable regions of Integron 1 and Integron 2

In the bacterial isolates containing the *IntI1* and the *IntI2* genes, the variable regions (VRs) of both of the studied integrons were sequenced in order to reveal the specific DNA sequence (plausibly bearing the multidrug resistance genes) within the integron structure. The regions were amplified with a set of specific primers: Integron CL (for integron 1) and Integron CL JJ (for integron 2), sequences are listed in Table 6. The PCR reaction conditions, and primer concentrations were the same as described for *IntI1* and *IntI2* amplification, with an annealing temperature of 61 °C. The amplified gene cassettes of similar PCR product length (base pair, bp) were sequenced by the Sanger method. Prior to sequencing, the PCR products were purified with FastAP and ExoI enzymes (EF0654 and EN0581, Thermo Fisher Scientific, Waltham, Massachusetts, USA), amplified With the BigDye™ terminator v3.1 Cycle Sequencing Kit (4,337,458, Life Technologies, Carlsbad, California, USA) and purified on Sephadex G50 (G5050, Sigma, St. Louis, Missouri, USA) by filtration. Sequencing of the cassette arrays was done with Applied Biosystems ABI 3130xl 16-capillary array genetic analyzer (Applied Biosystems, Waltham, Massachusetts, USA). Data was analysed with the Seqman software.

### Detection of antimicrobial resistance genes outside the integron cassettes

This experiment focused on the detection of 19 antimicrobial resistance genes in the studied genomic samples. Genomic DNA was extracted with Extractme DNA Bacteria Kit (EM02, Blirt, Gdansk, Poland) according to the manufacturer’s protocol. The PCR reaction conditions were the same as described for the detection of introns. Primer sequences are listed in Table 6. We tested for the presence of 4 genes associated with resistance to b-lactam antibiotics—ampicillin and amoxicillin (*bla*_SHV_, *bla*_TEM_, *bla*_CTX-M_, *bla*_OXA_*)*^61^, ciprofloxacin-resistance (*qnrA, qnrB, qnrS*)^62^, streptomycin-resistance (*strA-strB)*^63^, kanamycin-resistance (*aphA1*)^63^, gentamicin-resistance (*aac(3)-II*)^64^, sulphonamides-resistance (*sul1, sul2, sul3*)^63^, trimethoprim-resistantance (*dfr1, dfr5, dfr7/17*)^65^ and tetracycline-resistance (*tetA, tetB, tetC*)^63^. The analyses were done with the set of primers listed in Table 6 and the PCR reaction conditions were as described for the detection of integrons.

### Statistical analysis

The significance of the differences between presence and absence of integrons of class 1 and 2, and the predominance of class 1 integrons in relation to class 2 integrons were tested within each group (A, B, and C) using Pearson's Chi-squared Test. Relation between the presence of integrons and the phenotype of multi antibiotic resistance was tested with the use of Poisson Regression analyses where the dependent variable was the number of antibiotics, resistance, and the independent variable was the integron’s presence. These analyses were performed separately for integron class 1 and 2 and for each group (A, B, and C). All of the statistical analyses were performed with the use of the R environment^66^.
